# Massively Enlarged Wandering Spleen With Torsion and Infarction in a 10-Year-Old: Case Report and Comprehensive Literature Review

**DOI:** 10.1155/crpe/9927034

**Published:** 2025-08-06

**Authors:** Zaid Sawaftah, Omar Sawafta, Humam Emad Rajha, Ammar Hassouneh, Mosaikah D. Tawfiq Anati, Abdallah H. Hussein, Ahmad M. Abuayash, Haya Tariq Taha, Islam Rajab

**Affiliations:** ^1^Department of Medicine, An Najah National University, Nablus, State of Palestine; ^2^College of Medicine, QU Health, Qatar University, Doha, Qatar; ^3^Department of Radiology, Hebron Governmental Hospital, Hebron, State of Palestine; ^4^Department of Pediatric Surgery, Hebron Governmental Hospital, Hebron, State of Palestine; ^5^Department of Medicine, Al-Quds University, Jerusalem, State of Palestine; ^6^Internal Medicine Department, St Joseph University Medical Centre, Paterson, New Jersey, USA

**Keywords:** case report, pediatric surgery, splenectomy, splenic torsion, wandering spleen

## Abstract

**Introduction:** Wandering spleen (WS) is a rare condition characterized by abnormal splenic mobility due to congenital absence or acquired laxity of its suspensory ligaments. It is more prevalent in children and women of childbearing age and may present asymptomatically or with acute abdominal symptoms due to splenic torsion, which can lead to infarction or rupture.

**Presentation of Case:** We report a case of a 10-year-old female who presented with a 4-day history of persistent nonbilious vomiting, generalized abdominal pain, and fever. Physical examination revealed a rigid abdomen with tenderness and guarding in the right iliac fossa. Abdominal ultrasound demonstrated an ectopic, enlarged spleen (19 cm) in the lower abdomen, with absent blood flow on Doppler imaging. A contrast-enhanced computed tomography (CT) confirmed splenic torsion, showing the spleen in the mid-abdomen with twisted vascular pedicle and hypoperfusion. The patient underwent urgent splenectomy due to ischemic changes and infarction. Postoperatively, she recovered uneventfully and remained stable during follow-up.

**Discussion:** WS may be congenital, due to incomplete fusion of the dorsal mesentery, or acquired, associated with factors such as pregnancy or chronic splenomegaly. Splenic torsion is the most severe complication, requiring prompt intervention. Imaging modalities such as Doppler ultrasound and CT are critical for diagnosis. Splenopexy is the treatment of choice for viable spleens, whereas splenectomy is necessary for infarcted spleens.

**Conclusion:** WS is a rare, clinically challenging diagnosis requiring high suspicion. Early imaging and surgical intervention are essential to prevent life-threatening complications.

## 1. Introduction

Wandering spleen (WS), also known as splenoptosis or ectopic spleen, is a rare clinical entity characterized by abnormal splenic mobility due to congenital maldevelopment or acquired laxity of its suspensory ligaments. While WS predominantly affects children and women of childbearing age, its clinical presentation is highly variable, ranging from asymptomatic cases to acute abdominal emergencies. A significant complication of WS is splenic torsion, which can lead to infarction, rupture, and potentially life-threatening outcomes [1].

The clinical diagnosis of WS is inherently challenging, given its nonspecific symptoms, such as chronic or acute abdominal pain and the presence of an abdominal mass. Radiological imaging, particularly contrast-enhanced computed tomography (CT), is essential in confirming the diagnosis. Imaging findings typically include splenic displacement, evidence of vascular compromise, and complications such as torsion or infarction. Prompt diagnosis and timely intervention are crucial for optimal management. Surgical options vary depending on the spleen's viability, with splenopexy recommended for salvageable spleens and splenectomy reserved for infarcted or torsed cases [2].

This report details the case of a 10-year-old female presenting with an acutely torsed, massively enlarged WS resulting in infarction. By addressing the diagnostic and management challenges encountered, we aim to contribute to the growing body of literature and enhance clinical awareness of this rare but severe condition. The work has been reported in line with the SCARE criteria [3].

## 2. Case Presentation

A 10-year-old female presented to the emergency room (ER) with a 4-day history of persistent nonbilious vomiting and a 3-day history of generalized abdominal pain. She also reported fever, with a maximum recorded temperature of 38°C. There were no additional associated symptoms. Her past medical and surgical history was unremarkable, and there were no known drug or food allergies.

**Figure 1:**
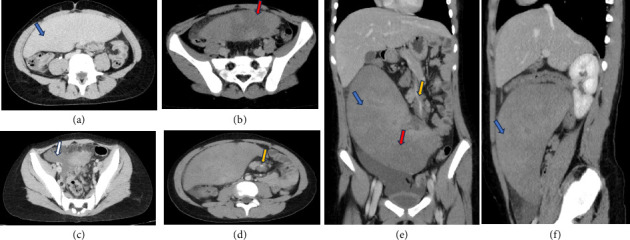
Abdomen CT with IV contrast in multiple planes, demonstrated: Absent spleen in the left upper quadrant; (a, e, f) massive enlarged spleen in the mid and lower abdomen (blue arrow); (b, e) hypodense heterogenous areas at the parenchyma of the spleen (red arrow) representing hypoperfusion, (d, e) whirled appearance of splenic vessels (yellow arrow); (c) pelvic free fluid (white arrow).

On physical examination, the abdomen was rigid, with marked tenderness in the right iliac fossa (RIF), rebound tenderness, and guarding. Given the patient's age, sex, and clinical presentation, initial differential diagnoses included intussusception, mesenteric adenitis, ovarian torsion, and gastroenteritis. Initial abdominal ultrasound revealed the spleen in the right lower abdomen, measuring significantly enlarged at 19 cm, consistent with a diagnosis of WS. Color Doppler imaging demonstrated absent blood flow in the splenic hilum, suggesting occlusion of the splenic vein and raising suspicion for splenic torsion. An urgent triphasic contrast-enhanced CT scan of the abdomen was recommended for further evaluation (Figure 1).

Laboratory evaluation revealed systemic inflammatory response (WBC 18.5 × 10^3^/μL, CRP 18.6 mg/L, ESR 45 mm/hr) with evidence of tissue ischemia (LDH 420 U/L, lactate 3.8 mmol/L). Coagulopathy (PT 19.2 s, INR 1.69) and thrombocytosis (838 × 10^3^/μL) were noted, along with mild hemolytic features (bilirubin 2.4 mg/dL). Metabolic acidosis (pH 7.32, bicarbonate 18 mEq/L) suggested systemic hypoperfusion (Table 1).

The CT scan confirmed the absence of the spleen in its typical anatomical position in the left upper quadrant and identified a massively-enlarged spleen located in the mid and lower abdomen. The spleen exhibited heterogeneous hypodense areas, consistent with hypoperfusion. Additionally, a whirled appearance of the splenic vessels was noted, indicative of vascular torsion. Free fluid was observed in the pelvis, further supporting the diagnosis of splenic torsion with associated complications.

The patient was admitted for urgent management. Initial interventions included keeping the patient nil per os (NPO), intravenous (IV) hydration with 2000 mL of pediatric saline, and analgesia with IV Paracetamol (350 mg, three times daily). Empiric antibiotic therapy was initiated with IV Cefuroxime (350 mg, three times daily) following a negative skin allergy test and IV Metronidazole (300 mg, three times daily). Two units of packed red blood cells (PRBCs) were cross-matched and prepared.

**Figure 2:**
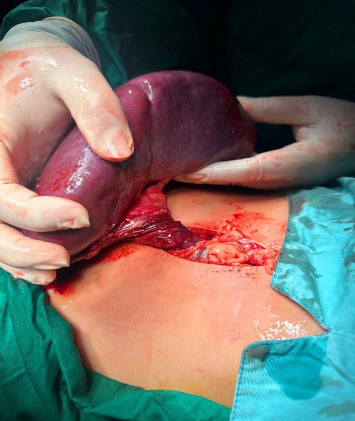
The image depicts an intraoperative finding of a wandering spleen. The spleen is visibly-enlarged and displaced from its normal anatomical position, with clear evidence of torsion at its vascular pedicle. The twisted pedicle suggests compromised vascular flow, which is consistent with splenic torsion. The surgical field is well-exposed, showcasing the extent of ischemic changes likely necessitating splenectomy to prevent further complications.

Given the duration of symptoms (4 days), imaging evidence of vascular occlusion (absent Doppler flow, whirled pedicle on CT), and hypoperfusion, splenic infarction was deemed irreversible. Intraoperative assessment confirmed a necrotic spleen with no salvageable parenchyma, necessitating splenectomy (Figure 2). Splenopexy was not attempted due to nonviability and the risk of post-torsion complications (e.g., thrombosis and delayed rupture). Postoperatively, the patient recovered uneventfully, tolerating oral intake by postoperative day 2. She received pneumococcal, meningococcal, and Haemophilus influenzae type b vaccinations prior to discharge to mitigate postsplenectomy infection risks. At the 3-month follow-up, she remained asymptomatic with no febrile episodes, and her parents were counselled on lifelong infection precautions (e.g., prompt antibiotic use for fever and malaria prophylaxis during travel).

## 3. Discussion

WS, also known as floating or ptotic spleen, is a rare condition primarily affecting children and women of childbearing age. Approximately one-third of cases occur in children, with 70% of these involving patients older than 10 years. The estimated incidence is less than 0.2%, with fewer than 500 cases reported in the literature [1].

WS occurs when the spleen is displaced from its usual left upper quadrant position to the abdominal cavity or pelvis. Normally, the spleen is anchored by the splenorenal, splenocolic, and gastrosplenic ligaments [2]. In WS, these ligaments are either congenitally absent or become excessively lax, resulting in a hypermobile spleen supported solely by its vascular pedicle. This predisposes the spleen to torsion, ischemia, necrosis, or rupture [4].

WS can be congenital or acquired. Congenital WS results from the failure of the dorsal mesentery to fuse with the posterior abdominal wall during embryogenesis and may be associated with anomalies such as diaphragmatic hernia or intestinal malrotation. Acquired WS is more common in women of childbearing age and is linked to factors such as multiple pregnancies, hormonal changes, splenomegaly (e.g., due to malaria or lymphoma), abdominal trauma, or prior surgeries [5].

Clinical manifestations range from asymptomatic presentations to life-threatening complications. Asymptomatic cases are often incidental findings during imaging [6]. Symptomatic WS may cause acute, intermittent, or chronic abdominal pain. Severe torsion can lead to acute abdominal pain, shock, peritoneal irritation, fever, nausea, vomiting, and weight loss. Compression of nearby structures may cause complications such as gastric outlet obstruction, uropathy, or portal hypertension [7, 8].

Diagnosis often begins with abdominal ultrasound, which can reveal splenic enlargement and absent vascular flow in torsion cases. Doppler imaging is critical for assessing vascular compromise. CT confirms the ectopic spleen and torsion, with findings such as the “swirl sign” indicating twisted splenic pedicle vessels. Additional features may include hypodense areas indicating hypoperfusion and a coma-shaped mass. Magnetic resonance imaging (MRI) is helpful in detecting thrombosis [9, 10]. Laboratory findings are nonspecific, though inflammatory markers may be elevated in complicated cases [7].

Management depends on spleen viability. For viable spleens, splenopexy is the treatment of choice and can be performed laparoscopically or via open surgery. In cases of infarction or rupture, splenectomy is necessary (Table 2). Early diagnosis and intervention are crucial to prevent serious complications [5, 10, 17].

## 4. Conclusion

WS is a rare condition with nonspecific clinical manifestations, making its diagnosis challenging. Early imaging is essential for accurate identification, as prompt recognition enables timely intervention. When the spleen remains viable, splenopexy is the preferred treatment, as it is minimally invasive and preserves splenic function. However, if splenic infarction or necrosis is present, splenectomy is necessary. Overall, early diagnosis and appropriate surgical management are critical for preventing severe complications and ensuring favorable outcomes in patients with WS.

## Figures and Tables

**Table 1:** Pertinent laboratory findings on admission.

Category	Test name	Normal range	Value	Significance
Inflammatory	White blood cells (WBC)	4.0 – 10.0 × 10^3^/μL	**18.5**	Leukocytosis
	C-reactive protein (CRP)	0–5 mg/L	**18.6**	Inflammation
	Erythrocyte sedimentation rate (ESR)	0–20 mm/hr	**45**	Inflammation

Hematology	Hemoglobin (HGB)	11.5–15.5 g/dL	**10.3**	Anemia
	Hematocrit (HCT)	36%–46%	**32**	Anemia
	Platelets	150 – 450 × 10^3^/μL	**838**	Thrombocytosis
	Red cell distribution width (RDW)	11%–15%	**16.5**	Anemia of inflammation

Coagulation	Prothrombin time (PT)	11–13.5 s	**19.2**	Prolonged
	INR	0.8–1.2	**1.69**	Coagulopathy
	D-dimer	< 0.5 μg/mL	**2.1**	Possible thrombosis

Chemistry	Lactate dehydrogenase (LDH)	140–280 U/L	**420**	Tissue ischemia
	Bilirubin (total)	0.2–1.2 mg/dL	**2.4**	Possible hemolysis
	Albumin	3.5–5.0 g/dL	**3.1**	Acute phase response

Electrolytes	Lactate	0.5–2.2 mmol/L	**3.8**	Hypoperfusion

Urinalysis	RBC (urine)	0–5/HPF	**8.3**	Hematuria
	Protein	Negative	Trace	—

Blood gas	pH	7.35–7.45	7.32	Mild acidosis
	pCO_2_	35–45 mmHg	38	—
	Bicarbonate	22–28 mEq/L	18	Metabolic compensation

*Note:* Bold values are significantly out of normal range.

**Table 2:** Summary of selected case reports in the literature.

Author and date	Patient	Complaint	Investigations	Management	Outcome	Notes
Wester and Co (2020) [11]	36-year-old female	1-day history of abdominal pain and vomiting. Diffuse abdominal tenderness.	Elevated lipase. Abdominal CT showed spleen in lower right quadrant.	Parenteral pain-control treatment and hydration.	Abdominal pain and vomiting were resolved. Uneventful recovery.	Splenectomy was deferred because evaluation for liver transplant was ongoing.

Hashiguchi et al. (2021) [12]	35-year-old female	Frequent left-sided abdominal pain a few months post-delivery	Contrast-enhanced CT showed spleen in middle of abdomen. Twisted of splenic artery and vein.	Laparoscopic splenectomy.	Abdominal pain resolved. Uneventful recovery.	Diagnosed with asymptomatic wandering spleen 20 years ago

Lebeul et al. (2021) [13]	34-year-old female	Acute abdominal pain in left flank. Splenomegaly.	Abdominal CT showed enlarged spleen in left flank and splenic vein stenosis complicated by segmental portal hypertension.	Detorsion and splenopexy in an extra-peritoneal pouch.	Abdominal pain resolved. Uneventful recovery.	

Schaeffer et al. (2021) [14]	37-year-old female	7 days of mild left upper quadrant abdominal pain with an acute severe left upper quadrant pain with nausea. Left upper quadrant tenderness without rebound or guarding.	Low WBC, hemoglobin, and platelets. Elevated lipase, AST, ALT, alkaline phosphatase, and bilirubin. Abdominal CT showed splenic volvulus with swirling of splenic vascular pedicle involving stomach and distal pancreas	Exploratory laparotomy, detorsion of spleen, and splenectomy	Abdominal pain resolved. Uneventful recovery.	

Poirier et al. (2024) [15]	39-year-old female	Persistent suprapubic abdominal pain for a week. Suprapubic mass	Abdominal-pelvic scan showed an ectopic spleen in the pelvis with whorl-like torsion of a splenic pedicle.	Laparoscopic splenectomy.	Abdominal pain resolved. Uneventful recovery.	History of congenital diaphragmatic hernia previously treated using a left subcostal approach.

Awan et al. (2019) [16]	35-year-old female	3 weeks of worsening intermittent colicky abdominal pain	Mild tenderness and no guarding in the left hypochondrium. Normal blood tests. Abdominal CT showed anterior displacement of the spleen and rotated splenic vascular pedicle. The stomach was indented by the displaced spleen and the tail of pancreas was located in sub-phrenic position	Laparoscopy performed. The spleen was not infracted but was congested and twisted around its long vascular pedicle. The torsion of the splenic pedicle was untwisted in a counter-clockwise direction. Splenopexy performed.	Abdominal pain resolved. Uneventful recovery.	

## Data Availability

The data that support the findings of this study are available from the corresponding author upon reasonable request.
